# Obstetrical Society of Philadelphia

**Published:** 1889-12

**Authors:** J. M. Baldy

**Affiliations:** Secretary


### OBSTETRICAL SOCIETY OF PHILADELPHIA.

Reported by J. M. BALDY, M. D., Secretary.

THURSDAY, OCTOBER 3, 1889.

Dr. THEOPHILUS PARVIN in the chair.

### REPORT OF A CASE OF TUBAL PREGNANCY, WITH SPECIMEN.—PROBABLE DIAGNOSIS, AND REMOVAL PRIOR TO RUPTURE.

BY THEOPHILUS PARVIN, M. D.

Mrs. Mary E. W——— was brought to the hospital of Jefferson Medical College, September 19th, suffering from a probable ectopic gestation. She is 26 years old, has been married seven years, and has had three living children, all of whom she has nursed, and during each lactation menstruation has regularly occurred; her youngest child is thirteen months old, and she is now nursing it. About the 29th of June, the usual flow began, but has continued ever since, with brief intermissions, under the use of medicines given by her physician, Dr. Horwitz. In the latter part of August, and in September, she has suffered somewhat from nausea; and this, with some other things, would have led her to believe she was pregnant, had not the hemorrhage from the uterus been so constant. About the 1st of September, she began to suffer with occasional violent attacks of pain low down in the left side, followed by much soreness; these attacks were especially liable to occur when she lifted her child.

Because of the temporary absence of Dr. Horwitz from the city, she went some three or four times to the dispensary of the Pennsylvania Hospital, where she was examined by Drs. Baldy and Bradford, who found a tumor on the left side of the uterus, tender and cystic, with a history of almost continuous bleeding for some weeks, there being, however, no signs of any shreds; pain in the lower part of the abdomen; only very meagre symptoms of pregnancy. A probable ectopic gestation was diagnosticated, and an operation urged.

The day that she came to Jefferson Hospital, she had, with the hemorrhage, a discharge of small, membranous fragments; whether decidual or not, of course, could only be certainly known by microscopic examination. A day or two before, some similar fragments were discharged, according to her statement.

Upon examination, I found a tumor adjacent to the uterus, upon the left side, the uterus somewhat enlarged, and very great sensitiveness to pressure, both in the vagina and in the lower part of the abdomen, especially at the left side.

The history, the examination, and the previous examinations of Dr. Baldy, with his conclusion, gave me little doubt that the case was one of tubal pregnancy.

Abdominal section was done on the 20th of September, Dr. Baldy kindly being present, and Dr. W. E. Ashton assisting in the operation. The gestation cyst included in the tube was removed, and the specimen is now shown you.

The patient's convalescence has, thus far, been uninterrupted, almost two weeks having elapsed since the operation.

### REPORT OF A CASE OF TUBAL PREGNANCY, WITH SPECIMEN.—NON-DIAGNOSIS, BUT REMOVAL PRIOR TO RUPTURE.—RECOVERY.

By J. M. BALDY, M. D.

Mrs. G. R— (colored) walked into the out-patient clinic of the Howard Hospital, July 5, 1889, suffering from pain in her abdomen, so similar to that which I have often seen go with a pyosalpinx, that I diagnosticated this disease before examining her. The examination revealed a large, apparently tortuous, tender mass, posterior, and slightly to the left side, giving a boggy sensation to the touch. The diagnosis was verified, a saline purge given, and an immediate operation advised.

One week later, a messenger summoned me to the home of the patient, where I found her lying on the bed, suffering from severe pain in the abdomen. She stated that the night before a fit of sneezing had given her a similar pain. She arose from the bed and walked to the table, a distance of about six feet. While standing there, she was seized with a severe, cramp-like pain, which doubled her up and lasted for a few moments. The idea took possession of me that she was possibly suffering from an ectopic gestation. I helped her on to the bed, and made a most careful examination, which revealed the following: She had been having children at regular intervals of about two years. She had a child just a year and a half before, since which time her menstruation had been pretty regular. With the exception of an occasional "pain in her belly," she had always been well. Before coming to me, she had been bleeding irregularly for about two weeks. The show was, and had been, clear blood, without any signs of shreds. Her husband had been away from home for about three weeks. The pain in the abdomen had been there since the bleeding began. There was not the slightest sign of pregnancy, either subjective or objective, nor did she think she was pregnant. A reëxamination of the pelvis showed only what had been before found, viz., a cystic mass, which did not pulsate, posterior and to the left—apparently a distended tube. The uterus was in position of normal anteflexion, and there was a perfectly normal cervix for a multipara. There was an elevation of temperature, and the woman had had chills and creeps. I asked Dr. Hamill to see the patient for me, and he verified my examination throughout. Together we decided that it was a case of pyosalpinx, stating, at the same time, that we had thought of ectopic gestation as a possibility, but were wholly unable to find sufficient data on which to verify our suspicion.

On July 13th, I opened the abdomen, with the assistance of Drs. Hamill and Naylor, in the presence of three or four gentlemen, and removed a left tubal pregnancy, which I here present to you. As I tore away the adhesions which bound the mass to the pelvic walls, the cyst was ruptured, and a teaspoonful of black clots was discharged from the sac itself. It was evidently a pregnancy of the fimbriated end of the tube, which had become adherent to the pelvic wall, the pelvic wall thus forming one side of the sac. The case well illustrates the difficulty, nay impossibility, of at times diagnosticating ectopic gestation. In three weeks the patient was sent home, and is to-day in her usual good health.

I would emphasize the following points, viz.:

The case is one of primary or unruptured tubal pregnancy. (There are now four such cases on record from this city alone, viz.: Dr. J. Price's, Dr. Goodell's, Dr. Parvin's, and my own.)

The patient is a colored woman, which is rather rare.

The patient did not have a long period of sterility, but was bearing children regularly.

There was at no time a sign of a decidual discharge.

There was at no time the slightest subjective or objective sign of pregnancy.

DISCUSSION.

Dr. E. W. CUSHING, of Boston.—The subject of extrauterine pregnancy is one of great interest to me, and I can say, from sad experience, that it is not easy to make a diagnosis. After some obscure symptoms of irregularity of menstruation, etc., a near relative was taken suddenly with a severe attack, which, after the event, I felt was due to a tubal pregnancy ruptured into the broad ligament; she finally recovered without operation. This turned my attention to the subject, and I looked up the specimens in the Harvard Medical School, which Dr. Parker photographed and I published. In another case, of which I saw the specimen, a gentleman operated for an ill defined tumor. The cyst was opened after the operation, and a fetus, three-fourths of an inch in length, found. There had not been a suspicion of pregnancy.

I believe that almost every one agrees in regard to the difficulties of diagnosis, and I believe that pretty much every one here agrees as to the necessity for surgical treatment; yet, as a subject for debate here, I would suggest, in opening this discussion, that there may be cases where a man may suspect extrauterine pregnancy, but yet be not sufficiently certain to operate, or not be able to get permission to do so, or he may be unable to do an abdominal operation himself, or secure the services of one that can. I would suggest that under such circumstances, the use of the faradic current is not only justifiable but prudent. This would be proper only in the earliest stages, before the fetus has reached such development that it would leave behind a source of irritation and suppuration. I think that the condemnation of the electric treatment in the early stage has been too sweeping and severe. Certainly the horrible cases which are recorded from attempting to puncture the fetal sac, especially at a later date, are not likely to be repeated.

Dr. WILLIAM GOODELL.—In regard to the electrical treatment of extrauterine fetation, I must confess that I was theoretically inclined to believe in it. But when I had met with cases of extrauterine fetation, and I saw the mass that was present, and the adhesions and injuries which adjacent organs had sustained, I could no longer uphold it. In my opinion, electricity should be reserved for those cases in which the woman absolutely refuses any surgical operation, or when the physician is not a laparatomist, and he cannot secure the services of one. The amount of adhesions is, however, so great, and the injury done the appendages so severe, that the woman cannot, in any case, conceive on that side. This was apparent in the case reported by me to the Society, in which I operated previous to the rupture. In this case, indeed, the appendages of the unimplicated side were so diseased as to need removal. The operation is, therefore, warranted, if, for no other reason, simply for the diseased tubes and ovaries. I have practically been converted to the belief that electricity, and particularly electrolysis, should not be used in these cases. The electrolytic action is a most dangerous one. Although advocated by Apostoli, the results have been most disastrous in the cases in which it has been tried.

Fifteen years ago, in a case which I now believe was one of extrauterine pregnancy, I punctured the tumor with an aspirating needle. In a few days septicemia set in, and the woman died.

The injection of morphia, as recommended by Winckel and by others, has met with better success. But while it destroys fetal life, it cannot cure the injuries sustained by the appendages, for which the best remedy is the knife.

I have had four cases of early extrauterine pregnancy within a few months, all of which were successful. One I supposed to be a simple case of disease of the appendages, and operated accordingly. It had burst, and without marked symptoms. In another case of ruptured sac, the specimen was perfectly analogous to that presented by Dr. Baldy, and he was present at the operation. The history of the third case I have already given to the Society, and I presented the specimen. I diagnosticated the ectopic pregnancy and operated before rupture. The fourth case was one of interstitial pregnancy, on which I operated the day before I went to Europe. The woman was brought to me by her physician, with the history of hemorrhages and great suffering, but with none of pregnancy. Her physician thought that the tumor was either a fibroid or a polypus. I found a fluid tumor bulging into the endometrium, and slightly dilating the os uteri. My diagnosis was a necrotic intramural fibroid tumor. Using Adam's subcutaneous saw, I cut into the mass, and removed a quantity of grumous blood and broken-down fragments. These latter were examined under the microscope and found to be placental tissue. The subject of extrauterine fetation is of great interest, not only before and after rupture, but also in relation to the cases that go on to term. I shall never forget the first case of extrauterine fetation that I saw. It was the classical case of the late Dr. Parry,—the one which led him to write his admirable essay on the subject.

A distinguished surgeon was called in. This was in the days when we made marked distinction between pelvic cellulitis and pelvic peritonitis. He diagnosticated the case as one of pelvic cellulitis. There was heat and great pain, with complete immobility of the womb. The patient did not improve, and I was called in, and diagnosticated the case as one of pelvic peritonitis, which, in one sense, it was, as the subsequent history will reveal. My treatment was not satisfactory, and I lost sight of the case. Several weeks afterward, while I was confined to my house for a few days by an illness, Dr. Parry came to see me. He sat down by my side, and after asking about my health, he referred to this case, and pointing his long forefinger at me, said, "You have made a great blunder." He then told me that he had been called in the day before to see the case, and that he considered it to be one of normal pregnancy, for he had heard the heart-sounds with the utmost ease; in fact, had never heard them so distinctly before. I said to him, "Dr. Parry, of course you are right in the theory of pregnancy; but depend upon it, there is something wrong, for I can hardly think that either Dr. ——— or myself could have made such a mistake without some good reason for it." The time for labor came, but it did not set in. Then the death of the child occurred. Dr. Parry was now again sent for, but this time he was unable to find the os uteri, so he sent for me. With great difficulty I found the cervix above the pubic bone. We then made the diagnosis of retroverted gravid uterus. At the next visit, it intuitively flashed across my mind that it was a case of extrauterine fetation, and then it became clear enough. To clinch the diagnosis, Dr. Parry introduced a hypodermic syringe needle and drew off some amniotic fluid. In a few days the head bulged down into the vagina, and we could distinguish even the sutures. We wished to incise the vagina and deliver the child with forceps; but the woman refused an operation, and died. At an autopsy Dr. Parry withdrew the sac and fetus, and exhibited them to this Society. Not long after this I saw a second case. The woman had passed full term, the child had died, and yet labor did not come on. Her physician, much puzzled, called me in. In this case the cervical canal was open, and, suspecting extrauterine pregnancy, I passed my finger into the uterine cavity and found it empty. The case was seen by one or two other physicians in consultation with us. We were anxious to operate, but the husband would not permit it, unless we could assure him positively that his wife would recover. While waiting, the woman suddenly died.

The third case I saw, a number of years ago, in a mulatto. The child was living at the time that I operated. There was no difficulty, either in the diagnosis or in the operation. The child, being hardly viable, gasped a few times and died. I did not dare to remove the placenta, and, as the umbilical cord was very large, I made the mistake of leaving it in the lower angle of the wound as a drainage-tube. The woman died a few days later, and at the autopsy we found the liver and lungs riddled with pyemic abscesses.

I operated on another case, in which the fetus must have died at the age of six months. The woman was perishing from blood-poisoning. She was emaciated, had high temperature, and night-sweats. Pus was evidently present somewhere, and I diagnosticated the tumor as a suppurating ovarian cyst. What perplexed me was great resonance in front. At the operation, this was explained by the presence of the gases of decomposition. Excessively fetid pus escaped. I removed the fetus, the bones of which, with the scalp and umbilical cord, were the only parts intact. The placenta was not to be found. The woman died suddenly from supposed heart-clot, after a violent altercation with her husband.

Not long after, I was called to Mount Holly to see a case which had been correctly diagnosticated by the physician. I removed a petrified seven-months' child. The patient had albuminuria, but did well until the eleventh day, when uremic convulsions set in, and she died comatose. All these operations were performed before the days of antiseptic surgery, and I have not since seen a case of advanced ectopic gestation.

I believe that Tait is correct in explaining these advanced cases by the rupture of the tube, and the escape of the unbroken gestation sac into the fold of the broad ligament. The behavior subsequently is precisely like that of an intraligamentary ovarian cyst.

In regard to early diagnosis, I should say that the most common symptom is arrest of menstruation for one or two periods, followed by irregular uterine hemorrhages. It is true that pelvic colic is a common symptom, but not so common as the other. But I do not know that it is necessary to make an absolute diagnosis; given a woman with the exacting symptoms of a suspected extrauterine fetation, who has a displacing-tumor on one side of the womb, are we warranted in operating merely to remove the tumor, whatever its nature? Do we not constantly, on less provocation, remove pelvic tumors, whose character is determined only by the operation? Instead of an extrauterine fetation, we may find pyosalpinx, or an ovarian abscess; but were we not in duty bound to perform the operation, even at the risk of an error in diagnosis?

Dr. Barton C. Hirst.—I was, some time ago, called to a case in consultation, which presented a clear history of extrauterine fetation: cessation of two periods, hemorrhage with the discharge of deciduous membrane, a distinct tumor to one side of the uterus, and the subjective signs of pregnancy, with swelling of the breasts, and vomiting. Dr. Hamill and myself urged operation, but the family being dissatisfied, we were discharged. Another physician was called, and Dr. Parish was consulted. He recommended the use of electricity, and a current was applied, with relief of the symptoms, and, I believe, complete cure of the patient. There may be a varicose vein in the broad ligament, which, having burst, may present all the signs of an extrauterine fetation after rupture of the sac. I have had two such cases; in one case, I opened the abdomen and found a blood-tumor in the layers of the broad ligament, with considerable blood in the peritoneal cavity. From the history and physical signs, I am quite sure that this was not an extrauterine pregnancy.

I saw, in consultation, not long ago, a fatal case of this kind after labor. The labor was a difficult one, and ended by craniotomy. There was rupture of a vein in the broad ligament. The bleeding was first between the layers of the broad ligament. This then ruptured into the peritoneal cavity, and the woman died. There was no rent in the uterine wall. Such cases might be mistaken for extrauterine fetation.

Dr. M. PRICE.—In most of these cases, all that we can make out is that there is something which should be removed; but, as to a distinct diagnosis of extrauterine pregnancy being made, I do not believe that it is done one time in ten. Dr. Parvin, in his case, would not have been surprised to have found distention of the tube from any cause. In a case which presented all the symptoms of uterine pregnancy, and where I expected to find this condition, I found a pair of large pus-tubes. I have seen twenty or twenty-five cases of extrauterine pregnancy, nearly all of them ruptured tubal pregnancies. It does not interest us a particle whether the cases were diagnosticated or not. There is trouble present of such a serious character that it does not become us to lose a single moment. Most of these cases come into the coroner's, and not the surgeon's, hands. Delay in operating is adding ten per cent. to our mortality. It is our duty to operate on the first indication, and, if we are mistaken, to thank God for the absence of so serious a condition.

Dr. JOSEPH HOFFMAN.—I have twice operated for extrauterine pregnancy, and did not find it. I operated once for something else, and found extrauterine pregnancy. The first case presented the signs of extrauterine pregnancy to even a more marked degree than that of

Dr. Hirst—coming on after a sterility of eight years, a retroverted mass, flooding, and violent pain. At the operation, I found two pus-tubes. She was pregnant and miscarrying, and completed it after the operation. The second case was almost on a par with this. In the third case, I operated for a pus-tube, and found extrauterine pregnancy. The trouble is, that these men who claim positive diagnosis, do it from a single case, which, though by no means certain at the time of operation, resolves itself into an absolute diagnosis when they come to publish it. It is the dreams and the nightmare of desire to publish something startling, which make the diagnosis. There are such a variety of conditions in the pelvis, giving rise to these symptoms, that it is impossible to say absolutely what we have. I have gone over Dr. Thomas's list of cases, and found that, so far as absolutely correct early diagnosis is concerned, it is worth nothing. In only one case, if my memory serves me, was there a *post mortem*. In three or four, in which death occurred, there was no *post mortem*; and in the others, where the current was used, the diagnosis, in most of them, was made after symptoms of rupture.

The treatment by the electric current is advocated by Reeve, who strangely falls into a fallacious argument. He says that if we use electricity in cases where there are symptoms of extrauterine pregnancy, and these symptoms disappear after the use of the current, why not call them extrauterine, as we do in other diseases where diagnosis is made, and a line of treatment adopted with good results, and illustrates the argument by the use of mercury in syphilis. If the effect of electricity was as well established as that of mercury in syphilis, the argument would be more logical. When we feel that rupture has occurred, electricity is a dangerous thing to tamper with. The principal danger is in delay. The longer the growth is allowed to continue, the greater the danger from adhesions, rupture, and complications which cannot be foreseen.

Dr. J. Price.—There are some interesting facts in connection with the history of this subject. It is curious that, a few years ago, a man with an experience of one doubtful case, should discuss the subject before the American Gynecological Society. It is also curious that the same man, with a single experience of a woman sterile five years, having pelvic pain, irregular menstruation, a delayed period for six days, and recurring attacks of pain, should claim to have killed the fetus of an extrauterine pregnancy by the use of electricity for ten *séances* of half an hour's duration each, on consecutive days. Then follows another man with a history of one case, and another in consultation. The man with an experience of one case used electricity, and the case passes into the hands of another, who writes to the first that he is going to operate. The first physician at once writes not to do it, as he has killed the fetus; while the operator already holds in his hands a large hydrosalpinx.

Dr. Baldy, in a recent paper in the New York *Medical Record*, lays down certain propositions, and makes the diagnosis easy in many cases. He has forgotten that many of these patients die, as if with a ruptured aneurism, before the woman suspects that anything is wrong. These cases go to the coroner. In the cases that reach the surgeon, the hemorrhage has not been so great.

It is interesting to refer, in this connection, to the cases of Dr. Edis and Dr. Bantock. Dr. Edis found, on one side, an extrauterine pregnancy, and on the other, a small ovarian cyst. Dr. Bantock found, on one side, an extrauterine pregnancy, and a pus-tube on the other. I have here an enormous tubal pregnancy, with rupture at the pavilion, and the abdomen filled with blood. On the other side was a beautiful hydrosalpinx.

One of the prominent cases on record, I saw a few hours before the operation. We agreed that there was a doubtful history of extrauterine pregnancy, but no prominence was given to this point. It might have been a small ovarian or dermoid cyst, or a large hydrosalpinx. When the abdomen was opened, every one expressed great surprise at the presence of tubal gestation. The same case has flourished over the world as a case of positive diagnosis. This is the case in which letters were written inviting different men to the operation; but the letters were written after the operation. This case will not bear close scrutiny. If we look over the transactions of the American Gynecological Society, we shall find that the men who have had the least experience speak most positively.

Dr. Noble.—Monday, a week ago, I removed an extrauterine pregnancy, which was rather unusual in the condition present. In my case, the woman had skipped the period in August. There was nausea and other symptoms of pregnancy. In September, a week before the sickness should have occurred, she began to flow freely; then had cramps, and fainted in the street, and afterward had fainting attacks. I saw her two weeks after the beginning of the flow. At one time, while in the water-closet, she passed what she thought to be a large clot. The opinion of her physician was that she had had a mis carriage.

I found a mass on the left side, pushing the womb to the right. I recommended an operation, which was not consented to for some days. In the meantime, the hemorrhage continued. The patient was seen by Dr. Kelly, and we agreed that it was almost certainly an extrauterine pregnancy. At the operation, I found blood in the peritoneal cavity. The blood was almost entirely clotted. The ovum was attached, not far from the uterus. The hemorrhage had taken place in the tube, and the clots had been forced out through the fimbriated extremity. On the other side there was a hydrosalpinx. She had also had the rectal tenesmus. She has done well since the operation.

Dr. B. F. Baer.—I wish to go on record as one who believes that it is as easy to diagnosticate extrauterine pregnancy as any other condition within the abdomen (as hydrosalpinx or pyosalpinx) positively; but the man who says he can make such a diagnosis positively, is an unsafe man. Dr. Taylor assisted me some five years ago in a case of extrauterine pregnancy in which the diagnosis was made. The fetus had advanced to the age of two and a half months, and the symptoms which have been mentioned were present. There was enlargement of the uterus, and I heard the placental bruit. I felt fluctuation in this sac, and was almost certain that I detected ballotment. I, however, operated in an unscientific way, cutting through the vagina. I got the embryo, but the patient died.

Dr. M. Price.—Did I understand Dr. Baer to say that he heard the placental bruit at two and a half months?

Dr. J. M. Baldy.—How did Dr. Baer detect ballotment at this early stage?

Dr. Baer.—I have heard this murmur in normal pregnancy at two and a half months. I believe that it is not yet clearly understood whether the murmur is placental or simply a uterine murmur. It is, however, due to pregnancy.

In regard to ballotment, given a sac filled with liquor amnii and an embryo dangling in it, I think that ballotment could be readily detected.

Dr. William Goodell.—Dr. Baer is right in regard to early ballotment. This is said to be detected much earlier in extrauterine fetation than in natural pregnancy.

Dr. J. M. Baldy.—I know there are cases on record, but I doubt the occurrence of ballotment in these. It could not possibly have occurred in such specimens as that presented by Dr. Goodell a few months ago, or those of Dr. Parvin and mine presented to-night. And so it has been with all the cases I have seen. Reference has been made several times to my paper in the *New York Medical Record*, in which I lay down three propositions: The first is, that in a certain proportion of cases of extrauterine pregnancy in the early stages, the diagnosis is easy and unmistakable. The second, that in a certain (quite large) proportion of cases, sufficient symptoms are present to more than warrant a diagnosis of extrauterine pregnancy, such a pregnancy not being present. The third, that in a certain other proportion of cases the symptoms, until rupture has occurred, are entirely wanting, or are of such a dubious character as to in no wise warrant such a diagnosis. These propositions have been abundantly sustained by the cases reported to-night. Cases have been reported in which the diagnosis was made and the condition found, and there are enough of these cases on record to show that the diagnosis can be made at times. Then we come to the second group of cases, in which the diagnosis is justified by the symptoms, but something else is found. This I sustained by cases of my own, and it has been more than abundantly substantiated by cases reported to-night by Dr. Goodell, Dr. Hirst, Dr. J. Price, Dr. M. Price, and Dr. Hoffman. Dr. Johnson, of Washington, has reported a similar case. The third proposition is also sustained by cases reported to-night (in addition to my own) by Dr. Hoffman, Dr. J. Price, and others. If there are well-substantiated cases in which the diagnosis was not made and extrauterine pregnancy found, and, again, if we operate for extrauterine pregnancy, the symptoms justifying such a diagnosis, and something else is found, I cannot see how any sensible man can claim that it is possible to make a positive diagnosis. I agree with Dr. Hoffman that Dr. Thomas's cases are utterly worthless from a diagnostic point of view; nor does Dr. Thomas himself hold that it is always possible. The men who claim that a positive diagnosis can be made have most signally failed. For instance, the case of Mann, of Buffalo, which went into the hands of Wylie, who operated and did not find ectopic gestation; the case which Buckmaster, together with other medical talent, treated with electricity, even to puncturing, I think, and finally pronounced it normally pregnant; the case of Kelly, of Philadelphia, the diagnosis of which case has to-night been denied by two eye-witnesses, as well as others.

It is noteworthy that the men who claim the most on this subject, have had the least experience.

---

PROF. S. B. HOWELL has resigned his chair in the University of Pennsylvania, in order to devote his whole time to his classes in the Medico-Chirurgical and Philadelphia Dental Colleges. The Board passed a vote of thanks to Professor Howell for his many years of excellent service. Edward D. Cope, one of America's foremost geologists, was elected to succeed him.—*Times and Register*.

